# Changes in authoritarianism before and during the COVID-19 pandemic: Comparisons of latent means across East and West Germany, gender, age, and education

**DOI:** 10.3389/fpsyg.2022.941466

**Published:** 2022-07-25

**Authors:** Ayline Heller, Oliver Decker, Vera Clemens, Jörg M. Fegert, Scarlett Heiner, Elmar Brähler, Peter Schmidt

**Affiliations:** ^1^Department of Psychosomatic Medicine and Psychotherapy, University Medical Center of the Johannes Gutenberg University Mainz, Mainz, Germany; ^2^Else-Frenkel-Brunswik-Institut, Leipzig University, Leipzig and Sigmund-Freud-University, Berlin, Germany; ^3^Department of Child and Adolescent Psychiatry/Psychotherapy, University of Ulm, Ulm, Germany; ^4^Department of Psychiatry and Psychotherapy, Leipzig University Medical Center, Leipzig, Germany; ^5^Centre for International Development and Environment (ZEU), Justus Liebig University Giessen, Giessen, Germany

**Keywords:** authoritarianism, second-order factors, measurement invariance, latent mean differences, repeated cross sections

## Abstract

Modern theories of authoritarianism have stressed the importance of threat to the expression of authoritarian attitudes and intolerance. Arguably, authoritarian tendencies may have increased during COVID-19 pandemic, a major threat to life and security. One issue arising when comparing mean scores is that of measurement invariance. Meaningful comparisons are only possible, if latent constructs are similar between groups and/or across time. This prerequisite is rarely ever tested in research on authoritarianism. In this study, we aim to analyze the short scale for authoritarianism KSA-3 by investigating its measurement invariance on two levels (three first-order and one second-order factors) and latent mean changes using two German representative samples (*N* = 4,905). Specifically, we look at differences before and during the pandemic (2017 vs. 2020). While measurement invariance holds across both levels in all conditions, we find a decrease in latent means in 2020, contrary to expectations and established theories. Moreover, latent means differ with regard to gender, education, and east–west Germany. We conclude that analyses of latent means and measurement invariance instead of mean comparisons with composites should become the standard. Future studies should focus on threat as a moderator between authoritarianism and intolerance, and on possible interactions with context variables.

## Introduction

The COVID-19 pandemic poses a massive, prolonging external threat to both life and health status of people all over the world as well as the global and local economy. Although Germany has managed the pandemic comparatively well, the consequences have been drastic. More than 4 million people were infected and over 90.000 people have died up to today in Germany (RKI – Robert Koch Institut, 2021). A lot of restrictions including two lockdowns took place (Steinmetz et al., 2020), which implied the closing of schools and child care institutions, restrictions in leaving homes and attending working places. Furthermore, the GNP as measure of total economic activity went down by 5% in 2020, which was a larger decline than after the financial crisis in 2007. It has been shown by the COSMO study, a weekly repeated cross-sectional survey, that this *objective threat* has been perceived by the German population as *subjective threat* as well (Betsch et al., 2020). Modern theories on authoritarianism suggest, that the expression of authoritarian attitudes and intolerance may increase in times of crises and insecurity (Oesterreich, 2005) and may modify its effect on outgroup rejection (Feldmann and Stenner, 2008). The challenging question remains, however, whether we are using adequate tools and techniques to compare levels of authoritarianism across time and across certain groups. In fact, it is conceivable that the pandemic may have altered the meaning of certain questions typically used or that the comprehension was different in certain groups to begin with.

To address this issue of *measurement invariance* as a precondition for all mean comparisons, we employed two representative cross-sectional studies, one before and one during/after the first lockdown, using identical instruments and data collection modes. Using the method of multigroup confirmatory factor analysis (MGCFA), we first tested the equivalence of meaning of the items and constructs over time. In addition, we examined whether this invariance also holds for the second-order factor authoritarianism, which explains the three subdimensions of authoritarianism: *authoritarian aggression, authoritarian submission and authoritarian conventionalism* (*cf.* Altemeyer, 1981, 1996). As second-order factor models have rarely been analyzed regarding their measurement invariance, we provide a systematic instruction (supplemented by an R-code as Supplementary Material) before comparing latent means. Finally, using the same approach, we analyzed differences in East and West Germany, age groups and educational groups. In contrast to comparisons with single items, summary or composite scores, all these analyses allow for a correction of random and non-random measurement error (Brown, 2015).

### Theoretical background

#### The theory of authoritarianism

Ever since its first conceptualization by Adorno et al. (1950), the idea of authoritarianism as an individual characteristic that promotes the development of anti-democratic attitudes as well as generalized and group-related prejudices has become a relevant research topic in different branches of the social sciences. It has been shown that authoritarianism is connected not only to right-wing voting behavior (Dunwoody and Plane, 2019), but also to the support of corporal punishment and violent educational methods (Clemens et al., 2019). It thus poses a threat to democracy and it may fire a cycle of violence by transmitting abusive behavior to the next generation (Clemens et al., 2020).

In its original formulation, authoritarianism was viewed as a stable character trait that was formed in early childhood due to socialization experiences in the nuclear family. In present times, the concept has been ridded of its psychoanalytic background. In the notion of *right-wing authoritarianism* (RWA), it is commonly treated as a set of attitudes rather than a personality trait (e.g., Altemeyer, 1981, 1996). Oesterreich (2005) saw the roots of authoritarianism in the lack of individual coping strategies to anxiety and insecurity. And indeed, many researchers could show that the endorsement of authoritarian attitudes is largely associated with personal, collective, and societal threat perception, causing authoritarian reactions to be dependent on time and situation (e.g., Duckitt and Fisher, 2003; Feldmann and Stenner, 2008). The ongoing COVID-19 pandemic poses a good example for this: The fear of infection combined with the restrictions of personal freedoms and limited options to psychologically process the drastic changes, authoritarian reactions could be registered throughout the world. A recent study by Golec de Zavala et al. (2020) reported an increase in authoritarianism and related attitudes in Poland using latent growth curve modeling on a representative, longitudinal data set. Moreover, Hartman et al., 2021 could show that anxieties about the pandemic increased the effect of authoritarianism on anti-immigrant attitudes in the United Kingdom and Ireland, and Deason and Dunn (2022) demonstrated that authoritarians were more likely to interpret the COVID-19 pandemic as a symbolic threat to their prevailing values than libertarians did.

Changes in authoritarian attitudes may also be attributed to differences in social systems, political socialization, interest and involvement (Duriez et al., 2005). With its history of division, Germany constitutes an interesting case to investigate the influence of these factors on authoritarian attitudes over time. In the past decades, the endorsement of authoritarian attitudes has been a lot stronger in those German states that were part of the former German Democratic Republic (GDR; Decker et al., 2020). Characteristics of the GDR itself as well as negative transformation experiences and persisting social inequalities between the Eastern and the Western states have all been taken into consideration as possible factors of influence (e.g., Best et al., 2014). In addition to temporal and regional trends, other important factors known to influence authoritarian attitudes are gender, educational background, and age. Regarding gender, women are less likely to endorse authoritarian attitudes than men, possibly caused by different socialization experiences (Rippl and Boehnke, 1995). Moreover, education is known to serve as a buffer for authoritarianism and other related attitudes (Heyder and Schmidt, 2000), and finally, younger people generally are less likely to express authoritarianism than older generations. It remains unclear whether the latter is due to older cohorts being socialized in more authoritarian environments (cohort effects), or a certain reluctance to change that is brought about by a psychosocial aging effect (Ruffman et al., 2016).

#### The issue of measurement

Measuring authoritarianism is an ongoing struggle. The first attempt to quantify authoritarian tendencies was the California F-scale developed by Adorno et al. (1950). The abbreviation is short for *Fascism* and points to the large overlap with right-wing extremist attitudes. It consists of nine dimensions and 30 to 38 items that were adjusted and revised multiple times (Adorno et al., 1950). The scale has been largely criticized for its item wording as it is heavily time- and culture-dependent. Moreover, the one sided answering format is likely to promote acquiescence, and social desirability threatens to further distort the results. Roghmann’s (2014) German translation of the scale tried to address some of these problems by constructing a balanced F-Scale with 11 minus items, taken from the original scale and 11 plus items, taken from other contemporary scales. Unfortunately, this scale has never been tested using representative data and thus, retest-reliability shows a wide range (0.17–0.85; Roghmann, 2014).

More recent scales draw on the theoretical and empirical developments by Altemeyer (1981,1996). The notion of RWA reduces the F-scale’s nine dimensions to three: *authoritarian aggression*, *authoritarian submission* and *conventionalism*. *Authoritarian aggressi*on captures a person’s tendency to devaluate and punish any socially deviant behavior. Respondents scoring high on *authoritarian submission* tend to look for the rule of a strong leader. Finally, *conventionalism* describes the adherence to established social norms and conducts. The dimensionality of his RWA scale is highly debated. Altemeyer considers authoritarianism to be a one-dimensional construct with three separate aspects. The scale does not clearly differentiate between these aspects though, with some of the 33 items showing double or even triple loadings in a factor analysis (see Hebler et al., 2014, for analyses on the German version of the scale). Additionally, some of the items show large overlap with related constructs that are often used as criterion variables, like group-related prejudice, religiousness, and conservatism, rendering it next to impossible to investigate the relationship between those constructs. The same holds true for most of the recently developed short scales.

Beierlein et al. (2014) propose a three-dimensional short scale with a second-order factor, the *Kurzskala Authoritarismus* (“Short Scale on Authoritarianism,” KSA-3). It is based on Altemeyer’s concept and addresses some of the shortcomings of previous scales: item wording shows little to no overlap with other constructs, factorial validity was assessed using confirmatory factor analysis (CFA) and correlation patterns with related constructs point toward construct validity. McDonald’s Omega as an indicator for reliability indicate adequate internal consistency (Beierlein et al., 2014). Finally, with only nine items (three per dimension), it easily allows for large scale usage, e.g., monitoring authoritarianism over long periods of time or in large, representative samples.

#### Measurement invariance

In this study, we aim to further examine the properties of this scale. In order to analyze differences in authoritarianism over time and across different groups, *measurement invariance* of these factors has to be investigated by testing the equivalence of constructs. This is needed in order to verify that differences between groups and changes over time are not due to arising differences in the comprehension of the questionnaires. The methodological literature has shown that comparisons between groups and time points might be severely biased when measurement invariance does not hold (Meredith and Millsap, 1992; Van de Vijver, 2018). For quantitative scales, CFA with multiple groups is an adequate tool to perform this task. Different levels of invariance are needed to verify certain assumptions about the construct across different groups and/or time. We generalize the results by Leitgöb et al. (2021) to second-order factor models and relate it to the Typology of Bias by Van de Vijver (2018).

*Configural invariance* is the least restrictive level as it imposes no equality constraints on the measurement model’s parameters, but only assumes the underlying factor structure to be equivalent across time and groups. In technical terms, the same pattern of factor loadings is expected to exist as a stable representation of the latent construct(s) by the indicators without the sudden absence of loadings or the occurrence of cross-loadings. If configural invariance is not met, construct validity as the central quality criterion of measurement and minimal requirement for any further analyses is not fulfilled (Leitgöb et al., 2021).

To establish the necessary condition for comparing the equivalence of concepts, full or partial *metric invariance* is needed, i.e., factor loadings over groups and/or time points are equal in at least two items of a given construct. This is the prerequisite of comparing variances and covariances and also regression coefficients over groups and/or time. Metric invariance additionally assumes that factor loadings of identical indicators are equivalent across groups and/or time. Varying factor loadings suggest that indicators differ in their relevance of defining the latent construct(s) under investigation. A lack of metric invariance thus affects the covariance structure among the latent construct(s) across groups and/or time, for example, it invalidates assessing the developmental stability of an underlying latent construct. Using additionally cognitive interviews, Meitinger (2017) and Van de Vijver (2018) show that these conditions are necessary but not sufficient to assume equivalence of the construct across groups and/or time.

*Scalar invariance* places further equality constraints on intercepts of identical indicators across time. Full or partial scalar invariance in addition to metric invariance is necessary to compare latent means and composite scores, i.e., item intercepts have to be equal in at least two items of a given construct. This precondition is much more difficult to reach.

These conditions can be generalized and are also valid for the relations between first-order factors and second-order factors like in our case of three first-order factors of authoritarianism and their relation to the second-order factor authoritarianism (Rudnev et al., 2018). In the typology of Van de Vijver (2018, p: 23) a lack of configural and metric invariance is related to construct bias, whereas scalar invariance is related to item bias.

As the political systems between East and West Germany differed massively in the years between 1945 and 1991, and even today regional differences in *per capita* income, unemployment, infrastructure, and many other factors remain (Bundesministerium für Wirtschaft und Energie – BMWi, 2020), it is necessary to test measurement invariance between these two regions in order to ensure that differences found in authoritarianism are not due to non-equivalence of constructs. Furthermore, due to many political events and changes that took place between 2017 and 2020—first and foremost the COVID-19 pandemic—it is unclear whether invariance can be assumed over time. To ensure that the changes in authoritarian attitudes brought about by the pandemic are not biased by a lack of equivalence in constructs, a test for measurement invariance is needed. If measurement invariance does hold, latent means can then be meaningfully compared.

## Materials and methods

### Participants

This study was conducted in the context of a regular, national representative survey of the general population of Germany. The data was collected by an independent institute for opinion and social research (USUMA) in 2017/18 (Sample 1) and 2020 (Sample 2). Inclusion criteria were an age ≥ 14 and the ability to understand the spoken and written German language adequately in order to understand and answer the questions. Informed consent was provided by all participants. At least one next of kin, caretaker, or guardian provided additional consent in case of minors. A multi-stage, random-route sampling procedure was applied to ensure representativeness: 258 sample points throughout Germany (210 in the former Western and 48 in the former Eastern states) revealed that 5,160 households should be contacted for Sample 1 and 5,418 for Sample 2. After exclusion of households that were vacant or without individuals meeting the inclusion criteria, 5,093 households (Sample 1) and 5,389 (Sample 2) were eligible for participation. Kish selection grid was then applied to select the target person within each household. Due to the COVID-19 pandemic, the participation rate was slightly lower in 2020 with 46.8% compared to 49.7% in 2017/18. The total sample size consisted of *N* = 5,034. After exclusion of those participants that did not completely fill out the questionnaire to be analyzed (*n* = 129), information on 4,905 participants was used for the final analysis (*n* = 2,465 for Sample 1 and *n* = 2,440 for Sample 2).

A sociodemographic interview was conducted by more than 200 trained and experienced interviewers for each sample. After the interview, participants had to fill out self-report questionnaires regarding political attitudes, physical and psychological symptoms. Interviewers were present but did not interfere unless there were questions. Table 1 gives an overview of the sociodemographic characteristics of the final sample as well as the two subsamples. The two samples did not show notable differences regarding these characteristics. Overall, the data can be assumed to be representative of the German population.

**Table 1:** Sociodemographic characteristics of the final sample stratified by year.

	2017/18	2020	Total
	*N* = 2,465	*N* = 2,440	*N* = 4,905
**Sex**	***N*** **(%)**	***N*** **(%)**	***N*** **(%)**
Female	1,369 (55.5)	1,290 (52.9)	2,659 (54.2)
Male	1,096 (44.5)	1,149 (47.1)	2,245 (45.8)
Missing		1	1
**Age**			
	***M (SD)***	***M (SD)***	***M (SD)***
Age, years	48.64 (17.88)	46.03 (17.73)	47.34 (17.85)
Age range	14–93	14–91	14–93
Age groups	***N*** **(%)**	***N*** **(%)**	***N*** **(%)**
14–29 years	465 (18.9)	546 (22.4)	1,011 (20.6)
30–39 years	355 (14.4)	400 (16.4)	755 (15.4)
40–49 years	388 (15.7)	365 (15.0)	753 (15.4)
50–59 years	511 (20.7)	524 (21.5)	1,035 (21.1)
60–69 years	421 (17.1)	373 (15.3)	794 (16.2)
≥70 years	325 (13.2)	232 (9.5)	557 (11.4)
**Relationship status**			
Married/living together	1,042 (42.3)	954 (39.1)	1996 (40.7)
Married/separated	63 (2.6)	69 (2.8)	132 (2.7)
Unmarried	795 (32.3)	966 (39.6)	1761 (35.9)
Divorced	336 (13.6)	290 (11.9)	626 (12.8)
Widowed	218 (8.8)	147 (6.0)	365 (7.4)
Missing	11 (0.4)	14 (0.6)	25 (0.5)
**Education**			
≤9 years	770 (31.2)	552 (22.6)	1,322 (27)
10 years	1,108 (44.9)	1,066 (43.7)	2,174 (44.3)
≥11 years	519 (21.1)	741 (30.4)	1,260 (25.7)
Still in school	60 (2.4)	74 (3.0)	134 (2.7)
Missing	8 (0.3)	7 (0.3)	15 (0.3)
**Employment status**			
Education/training	200 (8.1)	240 (9.8)	440 (8.9)
Working	1,319 (53.5)	1,360 (55.7)	2,679 (54.6)
Unemployed/working <15 h per week	204 (8.2)	223 (9.1)	427 (8.7)
Voluntary service, maternity leave, etc.	26 (1.1)	23 (0.9)	49 (1.0)
House wife/man	78 (3.2)	71 (2.9)	149 (3.0)
Retired	624 (25.3)	492 (20.2)	1,116 (22.8)
Missing	14 (0.6)	31 (1.3)	45 (0.9)
**Nationality**			
German	2,365 (95.9)	2,342 (96.0)	4,707 (96.0)
Non-German	100 (4.1)	90 (3.7)	190 (3.9)
Missing		8 (0.3)	8 (0.2)
**Income groups**			
0	146 (5.9)	173 (7.1)	319 (6.5)
0–500	93 (3.8)	123 (5.0)	216 (4.4)
500–649	78 (3.2)	65 (2.7)	143 (2.9)
650–749	122 (4.9)	86 (3.5)	208 (4.2)
750–899	150 (6.1)	125 (5.1)	275 (5.6)
900–999	145 (5.9)	109 (4.5)	254 (5.2)
1,000–1,149	158 (6.4)	139 (5.7)	297 (6.1)
1,150–1,249	186 (7.5)	131 (5.4)	317 (6.5)
1,250–1,499	347 (14.1)	289 (11.8)	636 (13.0)
1,500–1999	488 (19.8)	395 (16.2)	883 (18.0)
2000–2,499	275 (11.2)	331 (13.6)	606 (12.4)
2,500–3,499	146 (5.9)	246 (10.1)	392 (8.0)
3,500–4,999	61 (2.5)	246 (10.1)	144 (2.9)
5,000 +	14 (0.6)	43 (1.8)	57 (1.2)
Missing	56 (2.3)	102 (4.2)	158 (3.2)
**Religion**			
Protestant	920 (37.3)	626 (25.7)	1,546 (31.5)
Catholic	740 (30)	678 (27.8)	1,418 (28.9)
Muslim	60 (2.4)	114 (4.7)	174 (3.5)
Others	47 (1.9)	95 (3.9)	142 (2.9)
No confession	601 (24.4)	883 (36.2)	1,484 (30.3)
Missing	97 (3.9)	44 (1.8)	141 (2.9)
**Region of Germany**			
West	2014 (81.7)	1949 (79.9)	3,963 (80.8)
East	451 (18.3)	491 (20.1)	942 (19.2)

### Measures

The scale under investigation in the current study is the *Kurzskala Authoritarismus* (“Short Scale on Authoritarianism,” KSA-3) by Beierlein et al. (2014). It is a short scale designed to measure three dimensions of authoritarianism as proposed by Altemeyer (*aggression*, *submission* and *conventionalism*) using three items for each dimension. Participants are asked to indicate their opposition or agreement to nine items using a five-point Likert-Scale ranging from 1 = *I strongly disagree* to 5 = *I strongly agree*. Original wording as well as an English translation of the items may be found in Supplementary Material 1. Using CFA, the proposed factor structure showed an adequate fit and convergent validity with adjacent constructs indicated construct validity (Beierlein et al., 2014).

As to the sociodemographic factors, Table 1 shows that six *age groups* were differentiated. The oldest age group of 70 years and over was underrepresented compared to the general population, but all other age groups resembled the general populations’ percentage. As to the *relationship status*, 40.7% of the participants were married, 35.9% unmarried and 12.8% divorced. A smaller percentage was widowed (7.4%) or married but separated (2.7%). To make the results more accessible, *education* was converted into approximated years of schooling and split into four different categories. 27% of the participants had 9 years of schooling or less, 44.4% were in school for 10 years and 25.7% received 11 years or more (equivalent of a high school diploma). 2.7% of the participants were still in school. The majority of participants (54.6%) were working full time, 8.7% were currently unemployed. Only a small percentage (3.9%) reported a nationality other than German.

### Statistical analyses

To evaluate the psychometric properties of the KSA-3, we first assessed standard descriptive item statistics using IBM SPSS 24. Following the descriptive analyses, we aim to replicate the factor structure as tested by Beierlein et al. (2014) using CFA and assuming three first-order and one second-order factor. We report the fit for each point of measurement separately using the following cut-off criteria (Schermelleh-Engel et al., 2003): χ^2^ and χ^2^ divided by the degrees of freedom as well as the *Akaike Information Criterion* (AIC) should be as low as possible. Moreover, the *Comparative-Fit-Index* (CFI) and the *Tucker-Lewis-Index* (TLI) should score above 0.97 for a good fit and above 0.95 to be acceptable. Finally, both the *root mean square error of approximation* (RMSEA) and the *standardized root mean square residual* (SRMR) should fall below the cut-off of 0.05 for a good and 0.08 for an acceptable fit. Based on the CFA results, we report McDonald’s ω for each dimension stratified by time of measurement using the *semTools* package of the statistics software R version 3.6.1. Even though McDondald’s ω is more suitable for multidimensional scales than Cronbach’s Alpha (McDonald, 1999), we will be reporting the latter for comparative reasons. To assess the reliability of the second-order factor, we will be reporting ω_L1_ and ω_L2_. The former is conceptualized as follows: If a composite score were to be calculated from the observed indicators, ω_L1_ would describe the proportion of variance of that composite score that could be attributed to the second-order factor alone. It thus describes the reliability of the second-order factor at level one. The latter, ω_L2,_ on the other hand, can be defined as the reliability of the second-order factor at level two: If the first-order factors were calculated from error-free indicators, it would describe the proportion of variance of the first-order factors that could be attributed to the second-order factor.

To inspect measurement invariance, we used the *lavaan* package of the statistics software R version 3.6.1. We followed the steps suggested by Rudnev et al. (2018) that are depicted in Table 2. Five nested models are tested, increasing the model constraints with each step. For the configural model (1), all but one of the factor loadings, all item intercepts and all latent means of the first-order factor as well as all but one of the factor loadings of the second-order factor are freely estimated. The latent mean of the second-order factor is fixed to 0. For the first-order metric model (2), factor loadings of the first-order factor are set equal across groups with one factor loading per factor fixed to 1. In the first- and second-order metric model (3), the loadings of the second-order factor are set equal across groups in addition to that. In a fourth step, item intercepts of the first-order factors are set equal across groups and one per factor is fixed to 0. Finally, in the first- and second-order scalar model (5), latent means of the first-order factors are set equal across groups and latent means of the second-order factor are freed in all but one group. To evaluate whether or not invariance hold for each of the steps, we focus on the changes in CFI (Δ CFI) as proposed by Cheung and Rensvold (2002), because the χ^2^ difference test is known to be overly sensitive in large samples (>300). Δ CFI should not exceed 0.01. We also report the results of the χ^2^ difference test as well as changes in the other fit indices.

**Table 2:** Steps of measurement invariance in models with a second-order factor following Rudnev et al. (2018).

	First-order factors			Second-order factor	
	Factor loadings	Item intercepts	Latent means/intercepts	Factor loadings	Latent means
1. Configural	Free, but one per factor is fixed to 1	Free, but one per factor is fixed to 0	Free	Free, but one per factor is fixed to 1	Fixed to 0
2. First-order metric	Set equal across groups and one per factor is fixed to 1	Free, but one per factor is fixed to 0	Free	Free, but one per factor is fixed to 1	Fixed to 0
3. First- and second-order metric	Set equal across groups and one per factor is fixed to 1	Free, but one per factor is fixed to 0	Free	Set equal across groups and one per factor is fixed to 1	Fixed to 0
4. First-order scalar	Set equal across groups and one per factor is fixed to 1	Set equal across groups and one per factor is fixed to 0	Free	Set equal across groups and one per factor is fixed to 1	Fixed to 0
5. First- and second-order scalar	Set equal across groups and one per factor is fixed to 1	Set equal across groups and one per factor is fixed to 0	Set equal across groups	Set equal across groups and one per factor is fixed to 1	Free, but fixed to 0 in one group

If invariance does hold, we are then able to compare standardized and unstandardized latent means relative to the reference group using the final, first- and second-order scalar model. Values of *p* and standard errors are reported for each comparison.

## Results

### Descriptive item statistics

Descriptive statistics for each item stratified by the survey waves of 2017 and 2020 may be found in Supplementary Material 2. Since both skewness and kurtosis lay within the commonly agreed upon cut-offs of <2, we assumed normality for every item (Pituch and Stevens, 2016). We therefore refrained from using robust fit indices and used regular indices for the following analyses. Even though some missing values could be observed, they did not exceed the 5% mark. We thus assumed they did not significantly bias the results and decided to use the maximum likelihood (ML) estimator for all following CFA and MGCFA. Difficulty indices were consistently on a medium to low level, within the accepted span of 0.20 to 0.80, ranging from 0.37 (Item 6 in 2020) to 0.64 (Item 7 in 2017). Moreover, corrected item-total correlations scored on a high level, with each item above the cut-off of 0.40 (Moosbrugger and Kelava, 2012).

### CFA and reliability

A model with three first-order factors and one second-order factor was tested using CFA. From a statistical standpoint, a model assuming a second-order factor is identical to one that just consists of three first-order factors and thus leads to the same model fit (Brown, 2015). The assumption of a second-order factor stems from substantive theory: we assume that the three dimensions proposed by Altemeyer form a coherent, unitary construct (Beierlein et al., 2014).

**Figure 1:**
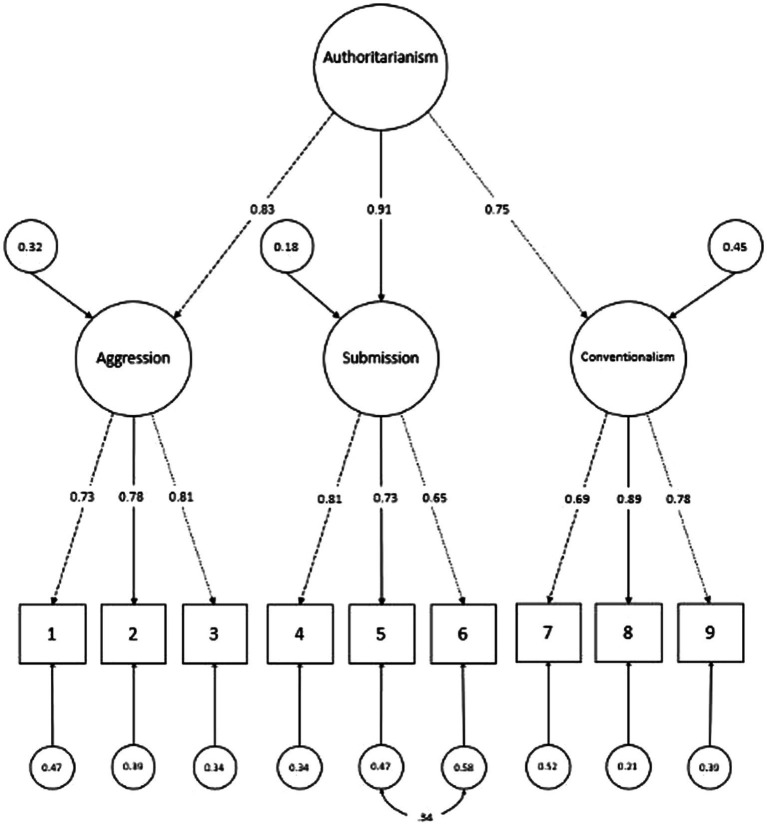
Structure tested in CFA and factor loadings for each dimension.

Modification indices suggested that letting the error terms of Item 5 and 6 covariates would lead to a large improvement of model fit. As Item 5 and 6 both belong to the same dimension, *authoritarian submission,* this adjustment seemed acceptable from a theoretical standpoint as well. Figure 1 depicts the structure of the final model and shows the standardized factor loadings on the first and second level. The latter may also be found in Supplementary Material 2, stratified by year. Factor loadings ranged from 0.63 for Item 6 on *authoritarian submission* in 2020, to 0.91 for *authoritarian submission* on the second-order factor in 2017 and overall. Mathematically, factor loadings in the CFA are comparable to item-total-correlation of classical test theory. While the latter is based on observed data, CFA takes into consideration the effect of other potentially correlated factors as the factor loadings are partialized regression coefficients, leading to more realistic estimations of the “true” values. In our case, connections between the items and their respective factors (or in case of the first-order factors: with the second-order factor) are consistently stronger than item-total-correlations (see Supplementary Material 2).

All indices pointed toward a good or acceptable fit with the pre-COVID, 2017 sample showing slightly better indices then the 2020 sample. Model fit indices for the final model stratified by year can be found in Supplementary Material 3. Internal consistency of the first-order factors ranged from 0.71 for *authoritarian submission* in 2020 to 0.85 for *conventionalism* in 2017. Internal consistency of the second-order factor was 0.77 at level one and 0.88 at level two, with the 2017 sample showing slightly better values than the 2020. McDonald’s omega and Cronbach’s alpha values for each first-order factor stratified by time of measurement as well as the second-order factor omega values at level one and level two are depicted in Supplementary Material 4. Based on these results, reliability of the scale can be judged as good to acceptable.

### Measurement invariance

Table 3 shows the results of the MGCFA that was used to test for measurement invariance over time as well as across the different regions of Germany, sex, age groups and education levels.

**Table 3:** Tests for invariance across time of measurement, region of Germany, gender, age groups and education.

	χ^2^ *(df)*	Δ χ^2^ (Δ*df*)	*p*	*CFI*	Δ *CFI*	*RMSEA*	*SRMR*	*AIC*
**Time of measurement**								
Model 1	609.837 (46)			0.974		0.071	0.031	118155.695
Model 2	628.259 (52)	18.422 (6)	0.005	0.974	<0.001	0.067	0.034	118162.117
Model 3	637.755 (54)	9.469 (2)	0.009	0.973	0.001	0.066	0.035	118167.613
Model 4	843.842 (60)	206.087 (6)	<0.001	0.964	0.009	0.073	0.040	118352.699
Model 5	855.621 (62)	11.779 (2)	0.003	0.964	<0.001	0.072	0.041	118369.479
**Region of Germany**								
Model 1	631.685 (46)			0.973		0.072	0.032	118292.687
Model 2	647.763 (52)	16.078 (6)	0.013	0.972	0.001	0.068	0.035	118296.764
Model 3	649.737 (54)	1.974 (2)	0.372	0.972	<0.001	0.067	0.035	118294.739
Model 4	682.805 (60)	33.068 (6)	<0.001	0.971	0.001	0.065	0.036	118315.806
Model 5	693.832 (62)	11.027 (2)	0.004	0.970	0.001	0.064	0.036	118322.834
**Gender**								
Model 1	622.723 (46)			0.974		0.072	0.031	118463.860
Model 2	636.855 (52)	14.132 (6)	0.028	0.973	0.001	0.068	0.034	118465.992
Model 3	637.602 (54)	0.747 (2)	0.688	0.973	<0.001	0.066	0.034	118462.739
Model 4	643.909 (60)	6.307 (6)	0.390	0.973	<0.001	0.063	0.034	118457.046
Model 5	647.985 (62)	4.076 (2)	0.130	0.973	<0.001	0.062	0.034	118457.121
**Age groups**								
Model 1	740.355 (138)			0.972		0.073	0.034	118325.681
Model 2	776.362 (168)	36.007 (30)	0.208	0.972	<0.001	0.067	0.038	118301.688
Model 3	787.372 (178)	11.01 (10)	0.357	0.972	<0.001	0.065	0.040	118292.698
Model 4	872.825 (208)	85.453 (30)	<0.001	0.969	0.002	0.063	0.042	118318.151
Model 5	947.464 (218)	74.639 (10)	<0.001	0.966	0.003	0.064	0.045	118372.789
**Education**								
Model 1	644.513 (69)			0.971		0.073	0.034	114375.363
Model 2	659.955 (81)	15.442 (12)	0.218	0.971	<0.001	0.067	0.036	114366.804
Model 3	665.075 (85)	5.12 (4)	0.275	0.971	<0.001	0.066	0.037	114363.925
Model 4	705.311 (97)	40.236 (12)	<0.001	0.970	0.001	0.063	0.039	114380.161
Model 5	747.924 (101)	42.613 (4)	<0.001	0.968	0.002	0.064	0.040	114414.773

df = degrees of freedom; CFI = comparative-fit-index; RMSEA = Root Mean Square Error of Approximation, SRMR = Standardized Root Mean-square Residual, AIC = Akaike’s information criterion, Model 1 = configural model, Model 2 = first order metric, Model 3 = first and second order metric, Model 4 = first order scalar, Model 5 = first and second order scalar.

Even though some of the χ^2^-differences were significant, *Δ CFI* remained below the cut-off of 0.01 in all conditions. As the χ^2^-test is known to be very sensitive to sample size, it is reasonable to assume measurement invariance over time, across regions of Germany, sex, age groups, and education levels. Latent means can therefore meaningfully be compared.

Table 4 gives an overview of the latent means relative to the respective reference groups. Latent means are significantly higher in 2017 compared to 2020, with a standardized latent mean difference of 0.225. Differences between East and West Germany are even larger, with the West exhibiting considerably lower standardized latent means. Regarding education, latent means show a gradient: when compared to the group with more than 10 years of formal education, the remaining groups show significantly lower latent means. Successively changing the reference categories suggested that there are significant differences between all three groups (see Supplementary Material 5 for detailed information). Finally, latent means of age groups show a very strong effect of old age. All other groups show significantly lower latent means when compared to the group of those ≥70 years and older. The effect seems to be equally strong for the youngest age group (14–29 years) but less pronounced for those groups in the middle.

**Table 4:** Estimated and standardized means.

	Latent mean (est.)	Standard error	Latent mean (std.)	*p*
**Time [Ref.: 2020]**				
2017	0.180	0.024	0.225	<0.001
**Region [Ref.: East]**				
West	−0.393	0.031	−0.536	<0.001
**Sex [Ref.: Female]**				
Male	0.060	0.024	0.082	0.011
**Education [Ref.: >10 years]**				
<10 years	0.553	0.034	0.783	<0.001
10 years	0.436	0.031	0.606	<0.001
**Age groups [Ref.: ≥70 years]**				
14–29 years	−0.466	0.046	−0.644	<0.001
30–39 years	−0.334	0.048	−0.468	<0.001
40–49 years	−0.239	0.047	−0.341	<0.001
50–59 years	−0.254	0.046	−0.337	<0.001
60–69 years	−0.284	0.047	−0.385	<0.001

Ref. = reference category.

## Discussion

The global threat of the COVID-19 pandemic has sparked renewed research interested in the concept of authoritarianism, as authoritarian tendencies seem to be on the rise as a reaction to perceived threat and insecurity. In this study, we set out to test the robustness of historic and current cleavages that have been considered to influence authoritarian dynamics in a society. To this end, we investigated measurement invariance across time and different social groups in order to confirm that latent means were indeed comparable and valid conclusions about possible causes and dynamics that lead up to authoritarian endorsement could be drawn.

Our results suggest that the short scale for authoritarianism, KSA-3, is indeed capable of reliably detecting mean differences across times and different groups. Descriptive item statistics showed no deviation from normality and CFA suggested a good model fit for a second-order factor model with three dimensions as first-order factors. McDonald’s Omega and Cronbach’s Alpha suggested good reliability for both the three first-order as well as the general second-order factor. Scalar invariance held for the first- as well as the second-order factors, suggesting that latent means are indeed comparable on both levels.

Contrary to our original hypothesis and established theories, latent mean comparisons showed lower scores of authoritarianisms in 2020 compared to 2017. There are different possibilities to account for these divergences: While Golec de Zavala et al. (2020) reported an increase in authoritarianism within the first months of the outbreak, our data covers a much longer time frame. It is thus possible that our study captures a long-term overall trend while Golec de Zavala et al. (2020) shed light on short-term fluctuations. In this regard, it is noteworthy, that in 2015 and 2016, there was a large refugee influx in Europe that lead to a widespread increase in xenophobic attitudes possibly affecting the 2017 survey. It is conceivable that this so called “refugee crisis” fired the authoritarian dynamic differently or more severely than COVID-19 pandemic. On one hand, there is a possibility that the perceived threat and insecurity caused by the pandemic has promoted other forms of coping. Øvretveit (2021), for example, reports innovations in self-care and close care made during the COVID-19 pandemic. Using digital technologies, fruitful adaptations and additions to existing social support systems were made that may have helped attenuate the impact of the crisis. On the other hand, conspiracy mentality has been reported to be on the rise in 2020 compared to 2018 (Decker et al., 2020)—a concept that formed one of the dimensions of authoritarianism of the F-Scale and has recently been re-integrated in the definition. Following Volkov (2000) idea of *cultural codes*, it is possible that authoritarian dynamics change their form of manifestation over time due to social norms and acceptability. As the threat of a virus may be more diffuse than that of refugees, the “scapegoat” may have shifted toward a more diffuse power as well. It is evident that the mere presence of an external threat does not suffice as an explanation for authoritarian tendencies. In fact, an interplay of policy making, political processes and subjective threat perception is quite conceivable (*cf.* Deason and Dunn, 2022) or that threat may only moderate the effects of authoritarianism on other constructs like outgroup rejection (Hartman et al., 2021) Cross-cultural and international comparisons as well as longitudinal studies could help shed light on this dynamic to understand how politics can promote solidarity in times of crises instead of fostering an authoritarian dynamic.

Measurement invariance across German regions suggests that previous findings of mean differences between East and West Germany are valid and should not be considered mere statistical artifacts that are due to a particular understanding of the questionnaire. There are several theories as to the nature of this divergence. Following Adorno et al. (1950), some researchers argue that the GDR socialization has led to a specific type of authoritarian character or disposition (Best et al., 2014) that is easily fortified by current situational factors. Others have focused on the widespread hardships of the transformation process and current, enduring inequalities between the East and the West as possible causes for dissatisfaction and frustration that would fuel the authoritarian dynamic. To untangle the specific effects and the interplay of historic and current factors that influence authoritarianism, future research should aim to differentiate between certain types of (post-)GDR-experiences as many scholars have criticized the trivialization and stereotypy of GDR-related research (*cf.* Haag et al., 2017).

To this end, an analysis of trajectories of different age groups or birth cohorts regarding their endorsement of authoritarian attitudes in East and West Germany could be a first step toward understanding the divergences. While our results suggest significant differences of both the oldest and the youngest age groups, the findings were less clear for the groups aged 30 to 69. As there may be a regional difference in cohort effects due to different socialization experiences, longitudinal analyses and/or more complex statistical approaches are needed in order to unravel the effects of age, time period and birth cohort on authoritarianism in the East and West.

Finally, we were able to confirm previous findings on the effects of education and sex: education has consistently been found to serve as a buffer for authoritarian and anti-democratic attitudes. There has been only little research on the exact mechanisms though. It is unclear whether the effect is enhanced by certain aspects of the school (e.g., class size, teachers, or content being taught) or whether it is a general, social aspect of being exposed to different world views. The same holds true for the differences between men and women: While some researchers have attributed the divergences to different socialization experiences (Rippl and Boehnke, 1995), Brandt and Henry (2012) take into consideration aspects of gender inequality that may lead to higher endorsement of authoritarian values in women in some societies, once again stressing the interaction of policy making and individual attitudes.

There are some additional limitations to this study. In the history of scale development, there has been a great debate about the problem of acquiescence when trying to capture authoritarian tendencies (Rokeach, 1967). While recent attempts to develop balanced short-scales have been reasonably successful with regards to the psychometric properties, there is still a risk of content overlap with criterion variables especially in the area of religiousness (*cf.* Heller et al., 2020). Another aspect, that could not be sufficiently addressed here, is that of cross-cultural validity. Perez and Hetherington (2014), for example, present evidence that authoritarian values may be judged differently in African American communities when operationalized as childrearing values, possibly influencing measurement invariance. Future studies should aim to validate and compare the various scales in different cultural contexts.

It is obvious that much more research needs to be done in order to unravel the interplay of individual and social factors influencing the authoritarian dynamic. Our study provides evidence that it is possible to analyze and compare mean scores across time and different groups using the KSA-3. Other scales should be analyzed in the same manner to ensure previous and future findings are indeed reliable. Such replications are extremely important to build up a more solid knowledge base in the social sciences, as it has been stressed by the discussions following the replication crisis and the creation of the Open science Forum and Movement and finally an Open Science Mind-Set (Hagger, 2022).

## Data availability statement

The data analyzed in this study is subject to the following licenses/restrictions: The data that support the findings of this study are available from the corresponding author upon reasonable request. Requests to access these datasets should be directed to AH, ayline.heller@unimedizin-mainz.de.

## Ethics statement

The studies involving human participants were reviewed and approved by University of Leipzig. Written informed consent to participate in this study was provided by all participants as well as a legal guardian/next of kin in case of minors.

## Author contributions

AH, EB, and PS contributed to conception and design of the study. OD, VC, JF, and EB organized the database. AH, SH, and PS performed the statistical analysis and wrote sections of the manuscript. AH and PS wrote the first draft of the manuscript. All authors contributed to the article and approved the submitted version.

## Funding

This research was funded by the Federal Ministry of Education and Research (BMBF) grant number 01UJ1911AY.

## Conflict of interest

The authors declare that the research was conducted in the absence of any commercial or financial relationships that could be construed as a potential conflict of interest.

## Publisher’s note

All claims expressed in this article are solely those of the authors and do not necessarily represent those of their affiliated organizations, or those of the publisher, the editors and the reviewers. Any product that may be evaluated in this article, or claim that may be made by its manufacturer, is not guaranteed or endorsed by the publisher.

## Supplementary material

The Supplementary Material for this article can be found online at: https://www.frontiersin.org/articles/10.3389/fpsyg.2022.941466/full#supplementary-material
