# Supplementary material for: Changes in authoritarianism before and during the COVID-19 pandemic: Comparisons of latent means across East and West Germany, gender, age, and education
Source: Front Psychol. 2022 Jul 25;13:941466. doi: 10.3389/fpsyg.2022.941466 (PMC9358451; doi:10.3389/fpsyg.2022.941466)
Supplement: Supplementary file 1 [file Data_Sheet_1.ZIP › Supplementary Material 1.docx]

Supplementary Material

# Supplementary Material 1

*Original item wording as well as English translation*

| Item 1 | Outsiders and under-performers in society should be severely punished | Gegen Außenseiter und Nichtstuer sollte in der Gesellschaft mit aller Härte vorgegangen werden. |
| --- | --- | --- |
| Item 2 | Troublemakers should clearly feel the effects of the fact that they are unwanted in the society. | Unruhestifter sollten deutlich zu spüren bekommen, dass sie in der Gesellschaft unerwünscht sind. |
| Item 3 | Social rules should be enforced without compassion. | Gesellschaftliche Regeln sollten ohne Mitleid durchgesetzt werden. |
| Item 4 | We need strong leaders in order to live safely in society. | Wir brauchen starke Führungspersonen, damit wir in der Gesellschaft sicher leben können. |
| Item 5 | People should leave important decisions to those in charge/the leaders. | Menschen sollten wichtige Entscheidungen in der Gesellschaft Führungspersonen überlassen. |
| Item 6 | We should be grateful for leaders who tell us exactly what we should do. | Wir sollten dankbar sein für führende Köpfe, die uns genau sagen, was wir tun können. |
| Item 7 | Traditions should absolutely be cultivated and maintained. | Traditionen sollten unbedingt gepflegt und aufrechterhalten werden. |
| Item 8 | Established conducts should not be questioned. | Bewährte Verhaltensweisen sollten nicht in Frage gestellt werden. |
| Item 9 | It is always best to do things in the usual way. | Es ist immer das Beste, Dinge in der üblichen Art und Weise zu machen. |
