# Supplementary material for: Changes in authoritarianism before and during the COVID-19 pandemic: Comparisons of latent means across East and West Germany, gender, age, and education
Source: Front Psychol. 2022 Jul 25;13:941466. doi: 10.3389/fpsyg.2022.941466 (PMC9358451; doi:10.3389/fpsyg.2022.941466)
Supplement: Supplementary file 1 [file Data_Sheet_1.ZIP › Supplementary Material 2.docx]

Supplementary Material

# Supplementary Material 2

| *Descriptive item statistics and standardized factor loadings* | | | | | | | | |
| --- | --- | --- | --- | --- | --- | --- | --- | --- |
| Item/Scale |  | *M* (*SD*) | Missings | Skewness | Kurtosis | *P^*^* | *r_it_^*^* | Factor loadings CFA (std.) |
|  |  |  | N (%) |  |  |  |  |  |
| Item 1 | Total | 3.02 (1.24) | 39 (0.8) | -0.03 | -0.91 | 0.51 | 0.58 | 0.73 |
|  | 2016 | 3.06 (1.28) | 15 (0.6) | -0.09 | -0.98 | 0.52 | 0.60 | 0.72 |
|  | 2020 | 2.99 (1.20) | 24 (1.0) | 0.02 | -0.83 | 0.50 | 0.57 | 0.74 |
| Item 2 | Total | 3.50 (1.22) | 44 (0.9) | -0.44 | -0.73 | 0.63 | 0.62 | 0.78 |
|  | 2016 | 3.51 (1.25) | 20 (0.8) | -0.50 | -0.71 | 0.63 | 0.67 | 0.82 |
|  | 2020 | 3.48 (1.18) | 24 (1.0) | -0.38 | -0.75 | 0.62 | 0.56 | 0.76 |
| Item 3 | Total | 3.13 (1.19) | 39 (0.8) | -0.09 | -0.80 | 0.54 | 0.67 | 0.82 |
|  | 2016 | 3.26 (1.23) | 16 (0.6) | -0.21 | -0.87 | 0.57 | 0.71 | 0.84 |
|  | 2020 | 3.00 (1.13) | 23 (0.9) | -0.01 | -0.67 | 0.50 | 0.62 | 0.77 |
| Item 4 | Total | 3.27 (1.24) | 32 (0.6) | -0.28 | -0.84 | 0.57 | 0.67 | 0.82 |
|  | 2016 | 3.33 (1.26) | 10 (0.4) | -0.32 | -0.86 | 0.58 | 0.70 | 0.85 |
|  | 2020 | 3.21 (1.22) | 22 (0.9) | -0.26 | -0.82 | 0.55 | 0.63 | 0.79 |
| Item 5 | Total | 2.67 (1.17) | 34 (0.7) | 0.18 | -0.78 | 0.42 | 0.65 | 0.73 |
|  | 2016 | 2.82 (1.21) | 16 (0.6) | 0.06 | -0.86 | 0.46 | 0.66 | 0.73 |
|  | 2020 | 2.53 (1.11) | 18 (0.7) | 0.28 | -0.65 | 0.38 | 0.62 | 0.72 |
| Item 6 | Total | 2.60 (1.16) | 33 (0.7) | 0.22 | -0.75 | 0.40 | 0.61 | 0.65 |
|  | 2016 | 2.72 (1.21) | 13 (0.5) | 0.12 | -0.85 | 0.43 | 0.64 | 0.66 |
|  | 2020 | 2.47 (1.10) | 20 (0.8) | 0.29 | -0.64 | 0.37 | 0.56 | 0.63 |
| Item 7 | Total | 3.52 (1.11) | 36 (0.7) | -0.40 | -0.49 | 0.63 | 0.58 | 0.69 |
|  | 2016 | 3.54 (1.15) | 18 (0.7) | -0.45 | -0.51 | 0.64 | 0.62 | 0.72 |
|  | 2020 | 3.50 (1.08) | 18 (0.7) | -0.34 | -0.46 | 0.63 | 0.54 | 0.65 |
| Item 8 | Total | 3.12 (1.18) | 39 (0.8) | -0.13 | -0.76 | 0.53 | 0.67 | 0.89 |
|  | 2016 | 3.25 (1.20) | 20 (0.8) | -0.22 | -0.76 | 0.56 | 0.70 | 0.91 |
|  | 2020 | 2.98 (1.14) | 19 (0.8) | -0.06 | -0.72 | 0.50 | 0.63 | 0.87 |
| Item 9 | Total | 2.81 (1.13) | 53 (1.1) | 0.09 | -0.64 | 0.45 | 0.62 | 0.78 |
|  | 2016 | 2.98 (1.15) | 34 (1.3) | 0.01 | -0.66 | 0.50 | 0.62 | 0.78 |
|  | 2020 | 2.64 (1.08) | 19 (0.8) | 0.13 | -0.63 | 0.41 | 0.60 | 0.78 |
| Authoritarian Aggression | Total | 3.22 (1.04) | 69 (1.4) | -0.18 | -0.59 | 0.56 | 0.61 | 0.83 |
|  | 2016 | 3.28 (1.09) | 30 (1.2) | -0.23 | -0.65 | 0.57 | 0.66 | 0.87 |
|  | 2020 | 3.16 (0.99) | 39 (1.6) | -0.14 | -0.52 | 0.54 | 0.55 | 0.77 |
| Authoritarian Submission | Total | 2.85 (1.02) | 56 (1.1) | 0.04 | -0.56 | 0.46 | 0.64 | 0.91 |
|  | 2016 | 2.96 (1.07) | 25 (1.0) | -0.03 | -0.64 | 0.49 | 0.67 | 0.91 |
|  | 2020 | 2.74 (0.97) | 31 (1.2) | 0.06 | -0.50 | 0.44 | 0.60 | 0.89 |
| Conventionalism | Total | 3.15 (0.98) | 73 (1.5) | -0.11 | -0.50 | 0.54 | 0.61 | 0.75 |
|  | 2016 | 3.25 (1.02) | 44 (1.7) | -0.18 | -0.54 | 0.56 | 0.63 | 0.75 |
|  | 2020 | 3.04 (0.93) | 29 (1.2) | -0.08 | -0.44 | 0.51 | 0.58 | 0.74 |
| *Note: P* = difficulty index, *rit* = corrected item-total correlation *based on N = 4,905 | | | | | | | |  |
|  |  |  |  |  |  |  |  |  |
