# Supplementary material for: Changes in authoritarianism before and during the COVID-19 pandemic: Comparisons of latent means across East and West Germany, gender, age, and education
Source: Front Psychol. 2022 Jul 25;13:941466. doi: 10.3389/fpsyg.2022.941466 (PMC9358451; doi:10.3389/fpsyg.2022.941466)
Supplement: Supplementary file 1 [file Data_Sheet_1.ZIP › Supplementary Material 3.docx]

Supplementary Material

# Supplementary Material 3

*Model Fit Indices of the CFA stratified by year*

| Model | N | χ ^2^ (df) | χ ²/DF | CFI | SRMR | RMSEA (90% CI) | TLI | | AIC |
| --- | --- | --- | --- | --- | --- | --- | --- | --- | --- |
| Total | 4,905 | 589.803 (23) | 25.644 | .974 | .034 | .071 (.066-.076) | .959 | 118,466.805 | |
| 2017 | 2,465 | 296.745 (23) | 12.902 | .978 | .032 | .069 (.063-.077) | .965 | 59,420.036 | |
| 2020 | 2,440 | 313.092 (23) | 13.612 | .969 | .037 | .072 (.065-.079) | .952 | 58,699.659 | |
