# Supplementary material for: Changes in authoritarianism before and during the COVID-19 pandemic: Comparisons of latent means across East and West Germany, gender, age, and education
Source: Front Psychol. 2022 Jul 25;13:941466. doi: 10.3389/fpsyg.2022.941466 (PMC9358451; doi:10.3389/fpsyg.2022.941466)
Supplement: Supplementary file 1 [file Data_Sheet_1.ZIP › Supplementary Material 4.docx]

Supplementary Material

# Supplementary Material 4

*Omega (and alpha) values of the dimensions*

|  | Authoritarian Aggression | Authoritarian Submission | Conventionalism | ω_L1_ | ω_L2_ |
| --- | --- | --- | --- | --- | --- |
| Total | .82 (.82) | .72 (.83) | .84 (.83) | .77 | .88 |
| 2017 | .84 (.84) | .73 (.84) | .85 (.84) | .80 | .89 |
| 2020 | .80 (.80) | .71 (.80) | .82 (.80) | .74 | .85 |
