# Supplementary material for: Changes in authoritarianism before and during the COVID-19 pandemic: Comparisons of latent means across East and West Germany, gender, age, and education
Source: Front Psychol. 2022 Jul 25;13:941466. doi: 10.3389/fpsyg.2022.941466 (PMC9358451; doi:10.3389/fpsyg.2022.941466)
Supplement: Supplementary file 1 [file Data_Sheet_1.ZIP › Supplementary Material 5.docx]

Supplementary Material

# Supplementary Material 5

## Table 1

*Differences in estimated latent means of authoritarianism based on education background using different reference categories*

|  | < 10 years | 10 years | > 10 years |
| --- | --- | --- | --- |
| < 10 years | - |  |  |
| 10 years | 0.117*** | - |  |
| > 10 years | 0.553*** | 0.436*** | - |

*Note.* * p < .05; ** p < .01 *** p < .001

## Table 2

*Differences in estimated latent means of authoritarianism based on age groups using different reference categories*

|  | 14-29 years | 30-39 years | 40-49 years | 50-59 years | 60-69 years | >70 years |
| --- | --- | --- | --- | --- | --- | --- |
| 14-29 years | - |  |  |  |  |  |
| 30-39 years | -0.132** | - |  |  |  |  |
| 40-49 years | -0.227*** | -0.095* | - |  |  |  |
| 50-59 years | -0.212*** | -0.081* | 0.015 | - |  |  |
| 60-69 years | -0.182*** | -0.050 | 0.045 | 0.031 | - |  |
| >70 years | -0.466*** | -0.334*** | -0.239*** | -0.254*** | -0.284*** | - |

*Note.* * p < .05; ** p < .01 *** p < .001
